# Survey of Red Fluorescence Proteins as Markers for Secretory Granule Exocytosis

**DOI:** 10.1371/journal.pone.0127801

**Published:** 2015-06-19

**Authors:** Nikhil R. Gandasi, Kim Vestö, Maria Helou, Peng Yin, Jan Saras, Sebastian Barg

**Affiliations:** Institute of Medical Cell Biology, Uppsala University, Box 571, Husargatan 3, 75123, Uppsala, Sweden; University of Edinburgh, UNITED KINGDOM

## Abstract

Fluorescent proteins (FPs) have proven to be valuable tools for high-resolution imaging studies of vesicle transport processes, including exo- and endocytosis. Since the pH of the vesicle lumen changes between acidic and neutral during these events, pH-sensitive FPs with near neutral pKa, such as pHluorin, are particularly useful. FPs with pKa>6 are readily available in the green spectrum, while red-emitting pH-sensitive FPs are rare and often not well characterized as reporters of exo- or endocytosis. Here we tested a panel of ten orange/red and two green FPs in fusions with neuropeptide Y (NPY) for use as secreted vesicle marker and reporter of dense core granule exocytosis and release. We report relative brightness, bleaching rate, targeting accuracy, sensitivity to vesicle pH, and their performance in detecting exocytosis in live cells. Tandem dimer (td)-mOrange2 was identified as well-targeted, bright, slowly bleaching and pH-sensitive FP that performed similar to EGFP. Single exocytosis events were readily observed, which allowed measurements of fusion pore lifetime and the dynamics of the exocytosis protein syntaxin at the release site during membrane fusion and cargo release.

## Introduction

Fluorescent proteins (FP) are extensively used as genetically encoded florescent tags [1,2] to address a multitude of biological questions. Their popularity is primarily due to the ease of labeling proteins of interest in living cells and organisms. Systematic development of novel and improved FPs has led to a palette covering the entire visible spectrum, but every FP comes with its own advantages and disadvantages. Newly developed FPs are usually benchmarked for brightness, photochemistry, photostability, cytotoxicity, pH- and ion sensitivity, as well as subcellular targeting accuracy with a fairly standardized panel of subcellular localization sequences [3,4,5]. However, the latter does not include specialized organelles such as synaptic vesicles or dense core secretory granules, organelles that are involved in regulated exocytosis and release of neurotransmitters and hormones.

Regulated exocytosis involves Ca^2+^- and SNARE-dependent fusion of secretory vesicles with the plasma membrane to empty its cargo [6,7], and FP-tagged markers are increasingly being used to study exocytosis in neuronal synapses [8] and even release of individual vesicles [7,9] by live cell microscopy. Since opening of the initial fusion pore during exocytosis leads to neutralization of the acidic pH of the vesicle interior, pH-sensitive FP’s such as EGFP or pHluorin have been particularly useful in this respect. Depending on the pKa of the FP, the fluorescence of the intact acidic vesicle is dim but visible (EGFP, Venus), or absent (pHluorin). PHluorin has a neutral pKa [8,10] and is widely used for studying synapse function [11,12,13,14,15,16,17], exocytosis and fusion pore behavior [18,19,20,21,22], endocytosis [23,24], receptor trafficking [25,26], and endosome function [27]. It is essentially dark in the intact acidic vesicle lumen and reaches >70% of its maximal brightness at neutral pH [8,28], which is useful to reduce background fluorescence and therefore increase the signal to noise ratio of the measurements [8,10]. EGFP and Venus have slightly more acidic pKa’s which lead to 2–3 fold fluorescence increases when acidic organelles are neutralized [9,19,29,30,31]. Vesicles labeled with these proteins remain visible even in the intact acidified state. If soluble vesicle cargo is labeled with such a FP, exocytosis leads to a rapid increase (flash) in vesicular fluorescence in the moment of fusion, followed by loss of the fluorescence as the cargo is released [9,30,32,33,34,35]. If instead a vesicle membrane protein is labeled, recycling and endocytosis processes can be studied [30,34,36,37].

For most other applications, it is desirable that FPs are insensitive to pH changes in the physiological range, and the development of novel FPs has consequentially focused on those with pKa <5. This has led to a paucity of pH-sensitive red FPs, as required for the study of exo- and endocytosis. Despite this, red FP’s such as mCherry [38] have been used for imaging secretory granule release in dual color applications. These essentially pH insensitive FPs (pKa<5) only report cargo release and cannot be used to study pore behavior during endocytic retrieval. Anecdotal reports also indicate that these FPs do not target as precisely as GFP derived markers, and that they may not report exocytosis as reliably as their green counterparts. More recently, several novel red FP’s have become available, some of which are pH-sensitive (pKa>6). These include mApple [5], mNectarine [39], mRuby2 [40], mOrange2 [5] and td-mOrange2 [41], td-tomato [38], pHred [42], pHtomato [43], pHuji and TYG-Cherry [28]. Their usefulness for the study of dense core granule exocytosis has not been explored systematically, although some have occasionally been used to image exo- or endocytosis [37,41,44]. Here we systematically survey available red FPs as tags for the study of dense core granule exocytosis.

## Methods

### Cells

**Fig 1:**
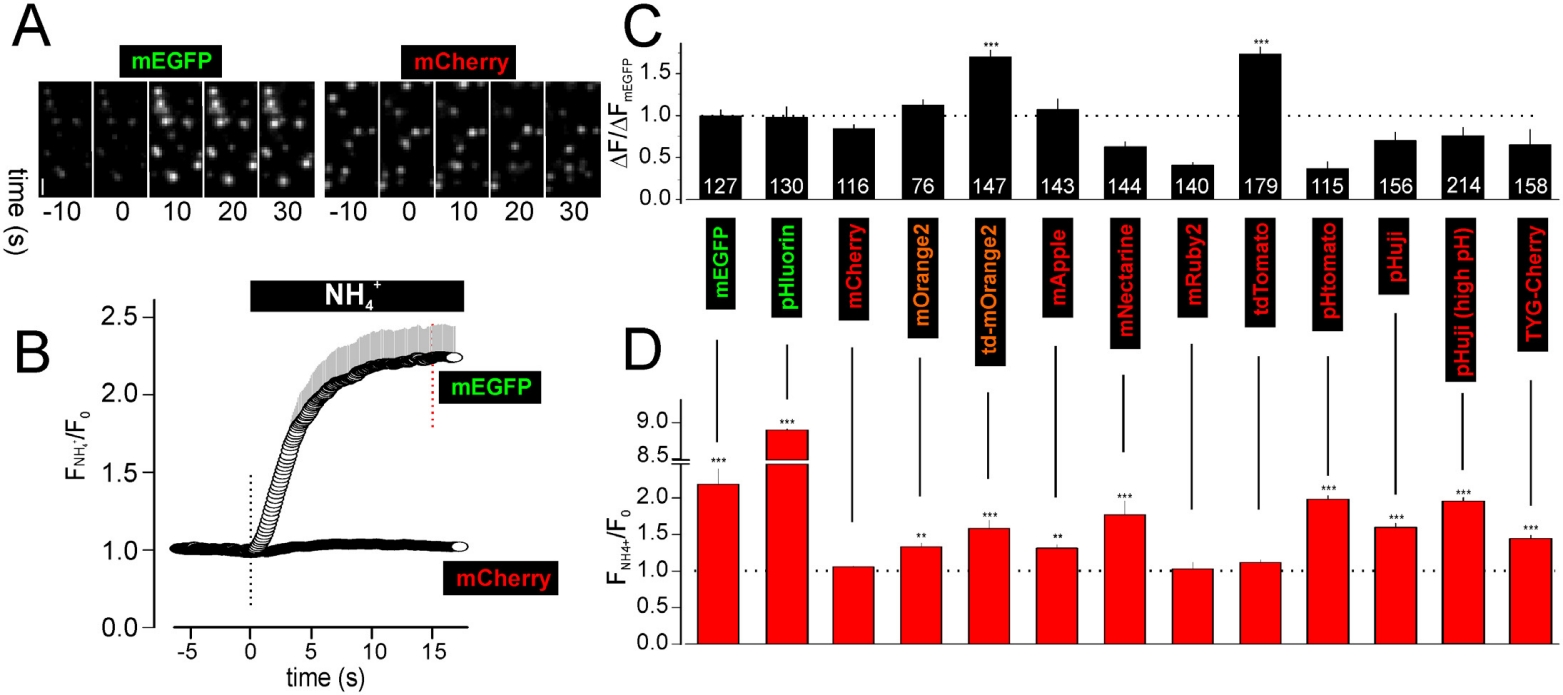
pH sensitivity of NPY-RFPs. To assess the pH sensitivity of FPs within intact granules, cells were transfected with NPY-FPs and exposed to 10 mM NH_4_Cl to neutralize the luminal pH. **A** Examples images of cells expressing NPY-mEGFP or NPY-mCherry. Times are relative to the onset of NH_4_^+^-exposure. **B** Fluorescence timecourse for experiments as in A (17 cells with NPY-mEGFP, 13 cells with NPY-mCherry). **C** Single granule brightness of NPY-FPs, in presence of NH_4_^+^ and normalized to NPY-EGFP (n of granules is shown on bars; from 35–76 cells each). **D** Average relative cellular fluorescence increase (F/F_0_) 15 s after application of NH_4_^+^ (12–20 cells each). Scale Bar = 1 μm.

**Fig 2:**
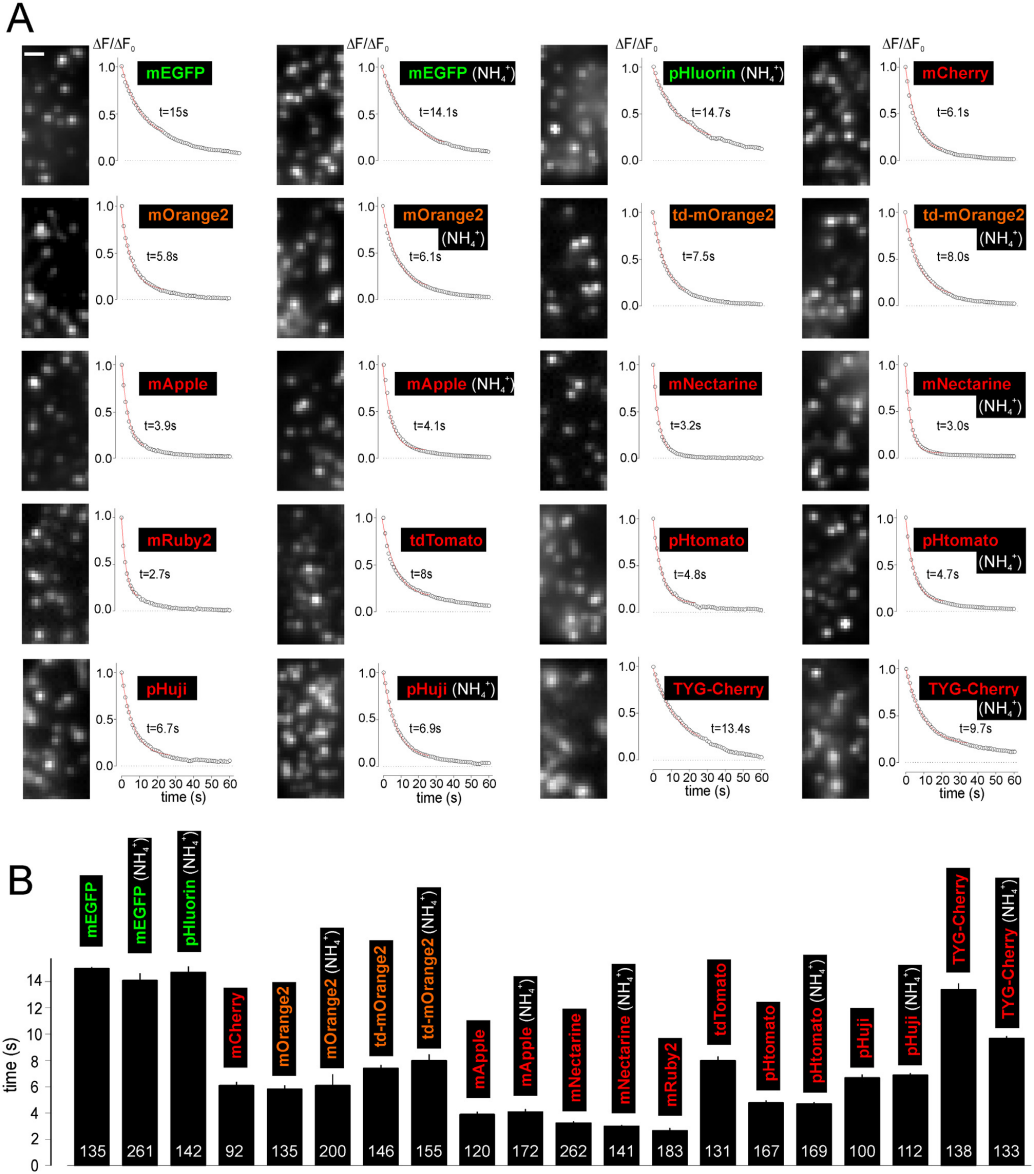
Photostability of NPY-RFPs. **A** Examples images and bleaching timecourse of NPY-FP transfected granules (ΔF/ΔF_0_). Imaging conditions were identical for all cells, except for excitation wavelength. Red lines are exponential fits used to derive the bleaching rate t. **B** Bleaching rates as calculated in A (n of granules is shown on bars; 8 to 19 cells each). Scale Bar = 1 μm.

**Fig 3:**
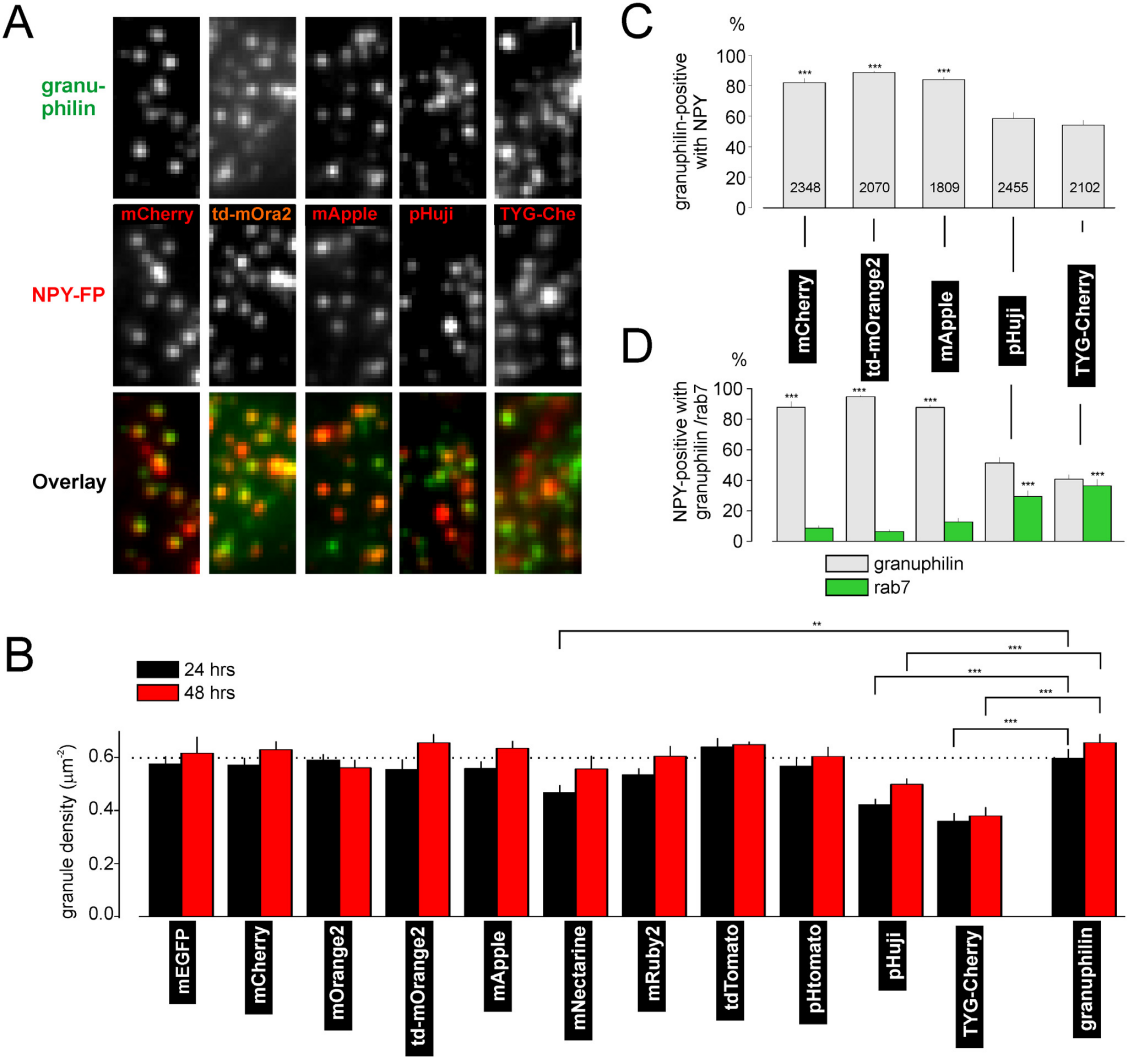
Most NPY-RFPs target well to secretory granules. **A** Example images of cells coexpressing EGFP-granuphilin and NPY-RFPs as indicated. **B** Quantification of granuphilin-positive granules in A that also express NPY-RFP (n of granules is shown on bars; 37 to 43 cells). **C** Quantification of NPY-RFP-positive granules in A that also carry granuphilin (grey, >300 granules from >20 cells each) or rab7 (green, >200 granules from 13 cells each). **D** Granule density observed with NPY-FPs or EGFP-granuphilin as indicated, 24 h (black) or 48 h (red) after transfection. (18–46 cells each).

**Fig 4:**
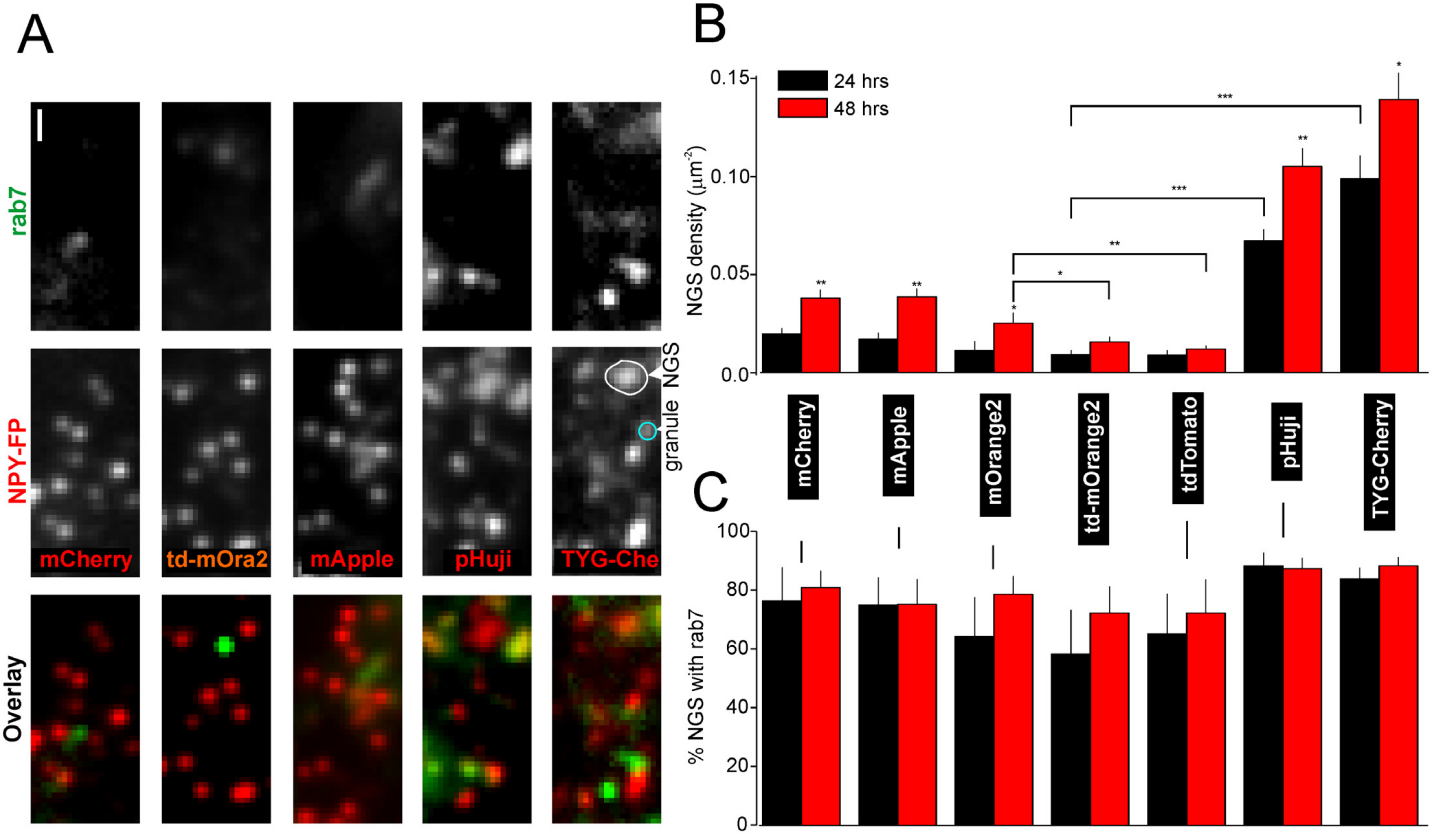
Mistargeting of NPY-RFPs to non-granular structures (NGS). **A** Example images of cells coexpressing EGFP-rab7 and NPY-RFPs as indicated. **B** Quantification of NGS density in A, 24 h (black) or 48 h (red) after transfection. (12–37 cells each). **C** Quantification of rab7 positive NGS in A. Scale Bar = 1 μm.

**Fig 5:**
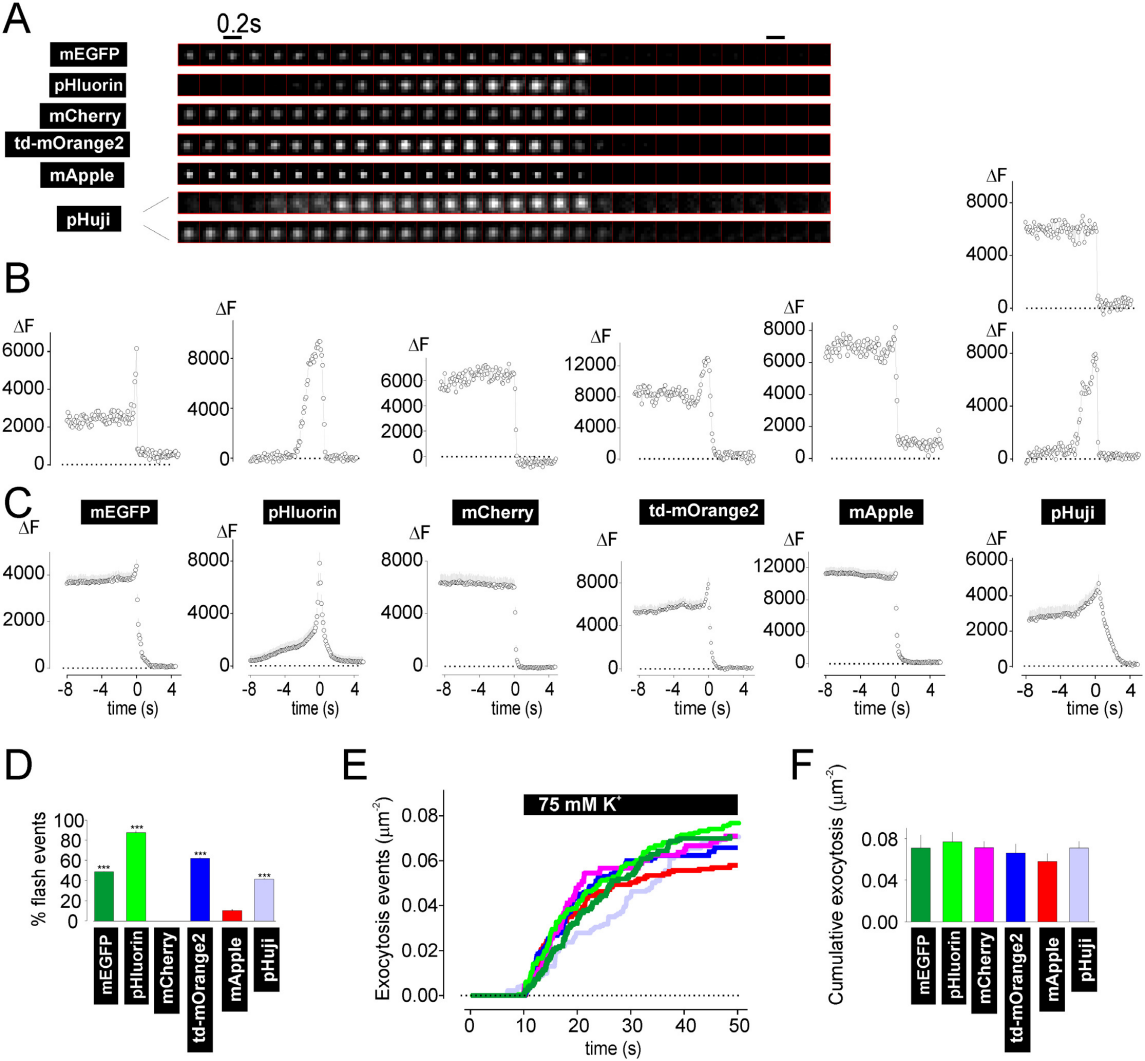
Visualizing exocytosis and release with NPY-RFPs. Exocytosis was evoked in cells expressing NPY-FPs by local application of elevated K^+^. **A** Examples of single granules undergoing exocytosis during stimulation. **B** Fluorescence timecourse of single exocytosing granules (ΔF). Note transient increase (flash) in many of the examples (46–96 granules, 8–11 cells). In case of pHuji the elevated K^+^-solution had pH 8.2; similar results were obtained at pH 7.4 (not shown). **C** Time-aligned averages of single granule exocytosis events as in A-B. **D** Fraction of flash events observed with different NPY-FPs. **E** Cumulative count of exocytosis events normalized to footprint area; timing of K^+^ stimulation as indicated. Traces are color coded for NPY-FPs as in D and F. **F** Total number of exocytosis events in E, normalized to footprint area. Scale Bar = 1 μm.

**Fig 6:**
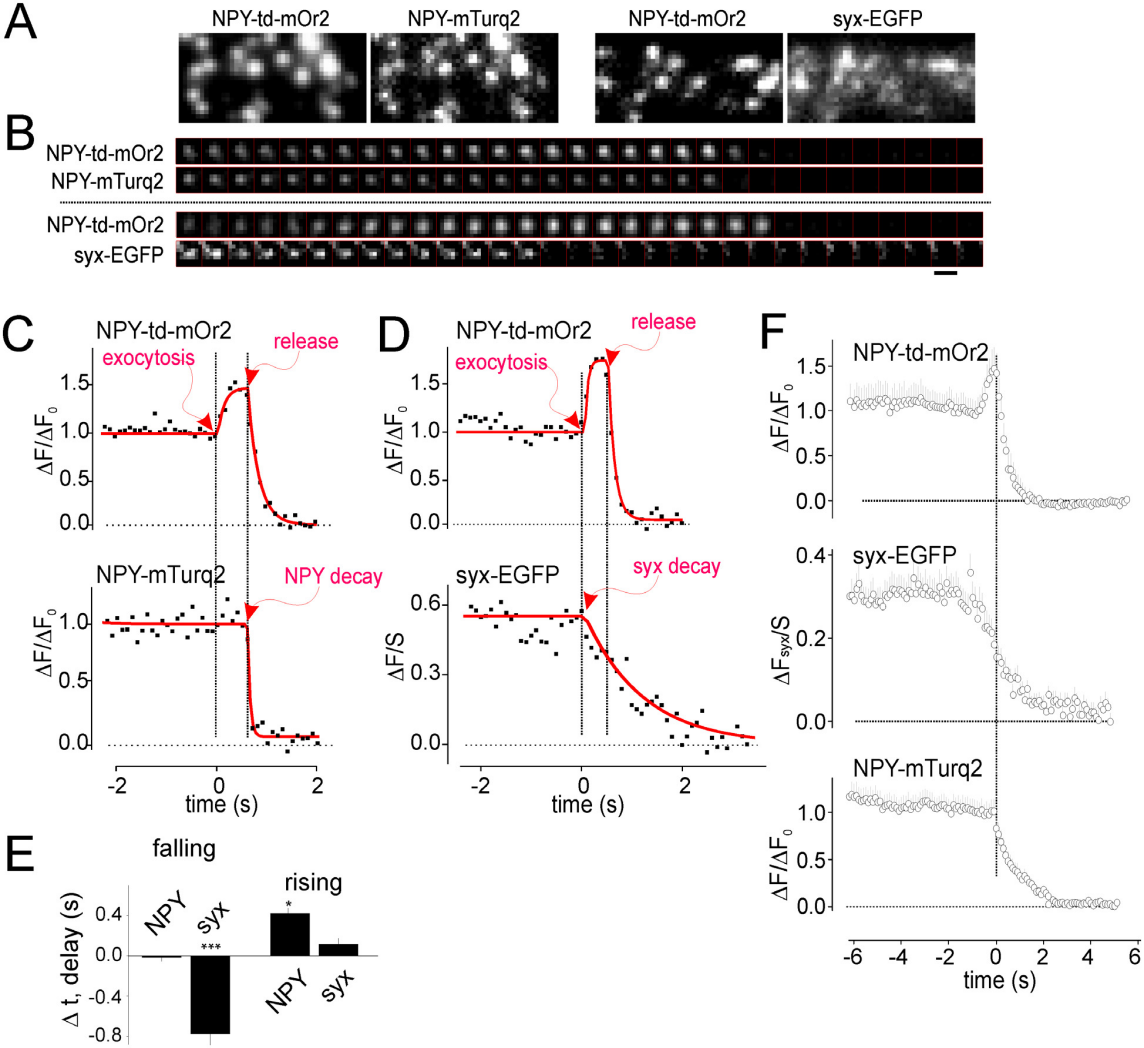
NPY-td-mOrange2 marks pore opening and release in double labeling experiments. **A** Example images of cells co-expressing NPY-td-mOrange with NPY-mTurquoise2 or syntaxin1A-EGFP. **B** Single exocytosis events in the double transfected cells. Note simultaneous release of NPY-td-mOrange2 with NPY-mTurquoise2 but not syntaxin-EGFP. **C-D** Time courses of single exocytosis events as in B. Red lines are non-linear fits as described in the text and methods. **E** Time delay between rising or falling phase of NPY-td-mOrange2 and onset of NPY-mTurquoise2 (NPY; 37 granules in 8 cells) or syntaxin-EGFP (syx; 48 granules in 10 cells) loss. Positive values indicate that the loss was initiated later than the reference event (rising/falling) in the NPY-td-mOrange2 channel. **F** Time-aligned averages of exocytosis events as in B. All traces were aligned to the falling phase of the NPY-td-mOrange2 signal. Scale Bar = 1 μm.

Ins1-cells was used as a model for testing dense core granule exocytosis; we used the clone 832/13 [45]. Cells were maintained in RPMI 1640 (Invitrogen) containing 10 mM glucose and supplemented with 10% fetal bovine serum, streptomycin (100 μg/ml), penicillin (100 μg/ml), Na-pyruvate (1 mM), and 2-mercaptoethanol (50 μM). The cells were plated on polylysine-coated coverslips, transfected using Lipofectamine2000 (Invitrogen) and imaged 24–34 hours later in Figs 1 and 2. In Figs 3D, 4B and 4C a set of experiments the specified cells were imaged after 48 hours. All other experiments in Figs 3, 4, 5 and 6 were performed 24–34 hours after transfection.

### Solutions

Cells were imaged in a solution containing (in mM) 138 NaCl, 5.6 KCl, 1.2 MgCl_2_, 2.6 CaCl_2_, 3 D-glucose, 5 HEPES (pH 7.4 with NaOH). To dissipate granule pH gradients, 10 mM NH_4_^+^Cl was added to this solution. For exocytosis experiments, glucose was increased to 10 mM and the solution supplemented with 2 μM forskolin and 200 μM diazoxide, a K^+^-ATP-channel opener that prevents glucose-dependent depolarization. Exocytosis was then evoked with high K^+^ solution (75 mM KCl equimolarly replacing NaCl). Test solutions (high K^+^, NH_4_^+^) were applied by computer-timed local pressure ejection through a glass pipette similar to those used for patch clamp. Exocytosis experiments were carried out at 32°C, all others at 22–25°C.

### Constructs

The constructs used were neuropeptide Y (NPY)-mEGFP [46], NPY-mCherry [38,47], and Syntaxin 1A-EGFP [30] (all obtained from W Almers), EGFP-granuphilin [48] (obtained from Addgene) and EGFP-Rab7 [49] (obtained from J Neefjes). NPY-pHluorin, NPY-mRuby2, NPY-mApple, NPY-mOrange2 and NPY-mTurquoise2 were generated by replacing mCherry at AgeI and NotI sites in NPY-mCherry with Age1/Not1 fragments of superecliptic pHluorin [8], mRuby2 [39], mApple [5], mOrange2 [5], or mTurquoise2 [50]. NPY-mNectarine and NPY-tdTomato were generated by replacing mCherry in NPY-mCherry with PCR fragments of mNectarine [39] (primers atccaccggtcgcaactatggtgagcaag and ttacttgtacagctcgtccatgc, AgeI and NotI sites) or tdTomato [38] (primers ataccggtcgccaccatggtgagcaaggg and ttacttgtacagctcgtccatgc, Age1 and BsrG1 sites). NPY-td-mOrange2 and NPY-pHtomato were generated by inverse fusion PCR cloning [51] to replace EGFP in NPY-EGFP with td-mOrange2 [41] or pHtomato [43] (primers tcgcggccgctttaacctgtgcctccgctctt, phos-gaccggtggatcccg, and agcactagcggcggaag or gtgagcaagggcgagg). NPY-pHuji was obtained by mutating K163Y using forward primer cggcgccctgtatagcgagatca, reverse primer phos-tcctcggggtacatccgct and NPY-mApple as template. NPY-TYG-Cherry was obtained by mutating M66T using forward primer cctcagttcacgtacggctcca, reverse primer phos-ggacaggatgtcccaggcg and NPY-mCherry as template. The same amount of DNA (0.5 μg/coverslip) was used for all fluorescent granule markers.

### Microscopy

Cells were imaged using a custom-built lens-type total internal reflection (TIRF) microscope based on an AxioObserver Z1 with a 100x/1.45 objective (Carl Zeiss). Excitation was from two DPSS lasers at 491 and 561 nm (Cobolt, Stockholm, Sweden) passed through a cleanup filter (zet405/488/561/640x, Chroma) and controlled with an acousto-optical tunable filter (AA-Opto, France). Excitation and emission light were separated using a beamsplitter (ZT405/488/561/640rpc, Chroma). The emission light was chromatically separated onto separate areas of an EMCCD camera (Roper QuantEM 512SC) using an image splitter (Optical Insights) with a cutoff at 565 nm (565dcxr, Chroma) and emission filters (ET525/50m and 600/50m, Chroma). Scaling was 160 nm per pixel. In Fig 1 cells were imaged at 100 ms per frame in stream mode with 491 (0.5 mW) for green FPs, 561 (0.2 mW) for mCherry, mOrange2, td-mOrange2, mApple, 561 (0.1 mW) for tdTomato, 561 (0.5mW) for pHtomato, pHuji and TYG-Cherry, 561 (1 mW) for mNectarine and mRuby2. In Fig 2 camera exposure was 200 ms per frame, with excitation 561 nm (10 mW) for red FPs and 491 nm (10 mW) for green FPs present throughout. The two color channels (Figs 3 and 4) were acquired sequentially, first with cells exposed to 491 nm (1 mW) for 1 s (50x20 ms average), immediately followed by 561 nm (0.5 mW) for 100 ms. In Fig 5 excitation was 100 ms per frame in stream mode at 561 nm (0.2 mW; 0.5 mW for NPY-pHuji) for red FPs and 491 nm (0.5 mW) for green FPs. In Fig 6 exposure was 100 ms per frame in stream mode with excitation simultaneously at 491nm (1 mW) and 561 nm (0.2 mW) or 405 nm (1 mW) and 561 nm (0.2 mW). Alignment of the two color channels was corrected as previously described [30].

### Image analysis

In Fig 1B–1D, the fluorescence emitted by the cell (F) was calculated as the pixel value in the footprint of the cell, corrected for out-of-cell background (bg). F is then normalized (FNH_4_^+^/F_0_) to the first frame of the movie (F_0_). All other measurements are specific for granules: Granules that were well separated from other granules or the edge of the cell were identified and an algorithm implemented as MetaMorph journal then read the average pixel fluorescence in 1) a central circle (c) of 3 pxl (0.5 μm) diameter, 2) a surrounding annulus (a) with an outer diameter of 5 pxl (0.8 μm) and 3) an area not containing any cell as background (bg). Since the granule site is far smaller than the resolution of the microscope, the circle will contain all of the fluorescence originating from it. To obtain the specific granule fluorescence ΔF, the annulus value (a) was therefore subtracted from that of the circle (c) (ΔF = c-a). In some cases, ΔF was normalized to the first frame of the movie (ΔF_0_) or the value obtained with NPY-mEGFP (ΔF_mEGFP_). Syntaxin was quantified similarly at granule sites (ΔF_syx_) and normalized with the off-granule syntaxin fluorescence (*S = a-bg*) [47]. *S* represents the local unbound concentration of syntaxin-EGFP. Positive ΔF/S values indicate binding of syntaxin-EGFP to the granule site, negative values indicate exclusion. Note that ΔF is given as per-pixel average for the entire 0.5 μm^-2^ circle, and ΔF/S values are therefore seemingly small. Assuming a cluster size of 50 nm, ΔF/S = 0.1 corresponds to at least 20-fold enrichment in the cluster beneath a granule.

Exocytosis events were found by eye as events with an obvious change in the fluorescence from the pre-exocytosis baseline and eventual rapid loss of the signal. This definition applied to both types of event, with or without preceding flash. The times of exocytosis and release were then obtained by non-linear fitting with a discontinuous function. The assumptions were (1) constant fluorescence before fusion, (2) inverted exponential decay just after fusion, and (3) exponential decay during content release (see Fig 6B and 6C). Fitting was carried out in Origin (OriginLab Corp, Northampton, MA, USA).

$$\begin{array}{l} c=A1+s1*\left(x-x1\right)\;\text{for}\;\text{t}<\text{t}1\\ c=A2-\left(A2-A1\right)e^{-\frac{x-x1}{t1}}\;\text{for}\;\text{t}2\;>\;\text{t}\;\geq \;\text{t}1\\ c=A3+\left(\left(A2-\left(A2-A1\right)e^{-\frac{x2}{t1}}\right)-A3\right)e^{-\frac{x-x2}{t2}}\;\text{for}\;\text{t}\;\geq \;\text{t}2, \end{array}$$ (1)

where t is time; c is average fluorescence in a 0.48 μm wide circle at the granule site; *A1*, *A2* and *A3* are the fluorescence values at the plateaus; τ1 and τ2 are the decay constants for the fluorescence increase after fusion and content release; t1 and t2 are the times of fusion and release, respectively, and *s1* is the pre-exocytosis slope.

Co-localization was estimated by an observer, as follows. A journal in MetaMorph presented an observer (unaware of the image context) with square cutouts of the green channel (11 μm^2^) that were centered on the position of each previously identified granule. The user then made a yes/no choice based on whether the center of the nearest perceived cluster was within one pixel of the center of the square, guided by an overlaid circle. Granule or non-granular structure (NGS) density was calculated using a script that used the built-in ‘find maxima’ function in ImageJ (http://rsbweb.nih.gov/ij) for spot detection.

### Statistics

Experiments were repeated with at least 3 independent preparations. Data are presented as mean ± SEM unless otherwise stated. One-way ANOVA was used for testing statistical significance in Fig 1. All the other data was tested for statistical significance using Students t-test for two-tailed, paired or unpaired samples, as appropriate. SigniFIcant difference is indicated by asterisks (*p < 0.05, **p < 0.01, ***p < 0.001).

## Results

### Brightness and bleaching of red fluorescent granule markers

We targeted a panel of ten red FPs or EGFP/pHluorin to insulin granules with the goal of comparing their brightness and bleaching rates. Fusions of the FPs with neuropeptide Y (NPY) were transfected in Ins1 cells. In all cases the footprint of the cells showed fluorescent puncta representing individual granules when imaged using TIRF microscopy (Fig 1A). Neutralization of the granule pH with NH_4_^+^ caused a rapid increase in granule fluorescence in case of EGFP but had no effect with mCherry (Fig 1A and 1C). In presence of ammonia, the granule brightness varied several-fold between red FPs with td-mOrange2 and td-tomato being brightest and mRuby2 and pHtomato being dimmest. Only the tandem dimers resulted in brighter granules (1.5-fold) than mEGFP or pHluorin (Fig 1C). The fluorescence increase observed with NH_4_^+^ was by far the strongest with pHluorin (~9-fold). The increase with mEGFP was >2-fold, td-mOrange2, mNectarine, pHtomato and pHuji about 1.5-fold, and mOrange2, NPY-mApple and TYG-Cherry about 1.3-fold. For mCherry, mRuby2 and tdTomato the changes were negligible, indicating pH-independent fluorescence in the physiological range (Fig 1D). Hence only td-mOrange2, mOrange2, mNectarine, mApple, pHtomato, pHuji and TYG-Cherry are sufficiently pH-sensitive to monitor the intravesicular pH change during granule neutralization. As expected from pHuji’s high pKa (7.6), it showed larger changes at extracellular pH 8.2 than at neutral pH (7.4) (Fig 1D). Transfection of granule-targeted pHred did not result in detectable fluorescence (not shown).

Fluorescence bleaching rates were measured in the absence of NH_4_^+^ during continuous exposure to excitation light and quantified by fitting with single exponential functions (Fig 2A and 2B). Bleaching was slowest for mEGFP (τ = 15 s) and pHluorin (τ = 14.7 s), followed by TYG-Cherry, pHuji, mCherry, td-mOrange2 and tdTomato (τ = 6–13.4 s). Worst were mNectarine, mRuby2, mApple and pHtomato (τ = 2.7–5 s). Considering that td-mOrange2 and tdTomato were twice as bright as mEGFP, reducing the excitation power would have resulted in brightness and bleaching behavior similar to mEGFP for both RFPs. Since the bleaching rate in the acidic environment in the granule might be different from that at neutral pH we repeated the measurements in the presence of ammonia (Fig 2A and 2B, NH_4_^+^). With the exception of NPY-TYG-Cherry, the bleaching rate was unaffected by the luminal pH.

### Most NPY-RFPs are reliable markers of dense core granules

Targeting accuracy of reporter proteins is important for many applications. We used the granuphilin tagged with EGFP (EGFP-granuphilin) as fiduciary granule label to quantify correct targeting of NPY-FPs. Since granuphilin is a soluble protein that selectively binds to granule membranes and is involved in granule tethering and docking [52], it labels the large majority of mature granules in insulin secreting cells. Indeed, the density of EGFP-granuphilin labeled granules was similar to previous estimates of granule density [53]. The density of labeled granules was close to that with granuphilin for most of the FPs tested, with the exception of mNectarine, pHuji, and TYG-Cherry where it was reduced by about a third relative to granuphilin (Fig 3B). About 80% of granuphilin-positive granules were labelled with NPY-mCherry, -td-mOrange2 or-mApple and 50–60% were labelled with NPY-pHuji or-TYG-Cherry (Fig 3A and 3C). Conversely, about 90% of NPY-mCherry, -td-mOrange2 and–mApple labeled puncta, and about 40–50% of NPY-pHuji or-TYG-cherry labeled puncta were granuphilin-positive (Fig 3A and 3D).

RFPs have been reported to be somewhat more cytotoxic than EGFP [54] and expression of some of the NPY-RFPs led to larger non-granule structures (NGS) that are unlikely to be granules (Fig 4A). We quantified mistargeting by co-expression with the endosomal marker EGFP-Rab7 [55,56] (Figs 3D and 4A–4C). With mCherry, td-mOrange2 and mApple fewer than 10% of all NPY-FP labeled structures co-localized with the endosome marker (Fig 3D). However, about 30% of NPY-pHuji and TYG-Cherry labeled structures co-localized with rab7 (Fig 3D). The density of NGS varied among the different RFPs used, and was lowest with td-mOrange2, slightly higher with mCherry and mApple, and strongly increased with NPY-pHuji and-TYG-Cherry (Fig 4A and 4B). The data suggest that tandem-dimer RFPs may be better tolerated than the monomeric FPs (Fig 4A and 4B). The reason why pHuji and TYG-Cherry target worse is not understood. It should be pointed out that most rab7-positive structures were large and most NPY-labeled NGS co-localized with rab7 (Fig 4A–4C), indicating their endosomal nature. Only about 2% of the small (diffraction limited) NPY-RFP positive structures carried EGFP-rab7. Small NPY-FP labeled structures therefore most likely represent secretory granules.

### Performance of NPY-RFPs for detection of single exocytosis events

Next we tested the performance of RFPs in reporting single granule exocytosis events. Exocytosis was evoked by local application of K^+^, which leads to depolarization and Ca^2+^-influx. After onset of the stimulation, exocytosis events were readily observed as sudden loss of granule fluorescence due to release of the label into the extracellular buffer. Cells expressing NPY-mEGFP and NPY-mCherry served as pH-sensitive and-insensitive references. With mEGFP, but not mCherry, this loss of fluorescence was often preceded by a temporary increase in fluorescence (flash), which we interpret as the opening of the fusion pore and concomitant change in luminal pH [9] (Fig 5A–5C). With NPY-mEGFP both flash and non-flash events were observed, presumably because pore opening and release can occur simultaneously. With both td-mOrange2 and mApple both types of events were observed. Flash events were somewhat more frequent with td-mOrange2 than with mEGFP, possibly reflecting the differences in their sensitivity to pH changes. The all-events average (Fig 5C) shows a notable transient increase with td-mOrange2, similar to what is observed with mEGFP. In contrast, this was hardly noticeable with mApple (Fig 5A and 5D), likely due to the fact that flash events were rare (Fig 5D). As expected from their pKa’s, pHluorin and pHuji resulted in flash events where the granules were not visible before exocytosis (Fig 5B). However, with pHuji about half of the events lacked the flash (upper example in Fig 5B, pHuji), consistent with the relatively small changes observed with ammonia (Fig 1). The total number of exocytotic events was similar for all FPs tested (Fig 5E and 5F). Large NGS (*cf*. Fig 4) never underwent exocytosis.

### NPY-td-mOrange2 reports exocytosis and release in dual color applications

Red granule markers are compatible with the large number of existing green FP-labeled proteins for dual color experiments. To demonstrate the usefulness of this we co-transfected NPY-td-mOrange2 together with NPY-mTurquoise2 (Fig 6A); both labels co-localized well. mTurquoise2 has a pKa of 3.1 and should not respond to physiological pH changes [50]. Indeed, the mTurquoise2 signal did not change during the rising phase of the td-mOrange2 signal, but both proteins were lost simultaneously during K^+^-evoked exocytosis (Fig 6B and 6C). To formally test what phase of the td-mOrange2 flash coincided with loss of mTurquoise2, a tri-part discontinuous function was fit to the individual traces (Fig 6C, red lines). From this we obtained the times of rising and falling phases (arrows) and calculated delays relative to these reference events. The analysis confirms that NPY-mTurquoise2 is lost simultaneous with the falling phase of the NPY-td-mOrange2 flash (Fig 6E). The results corroborate that the rising phase is due to luminal pH change rather than granule movement in the z-axis, and the falling phase due to content release.

We next analyzed cells co-expressing NPY-td-mOrange2 and syntaxin1a-EGFP. Syntaxin is a plasma membrane SNARE protein that lies at the core of the exocytotic machinery [47,57,58]. It is well-established that syntaxin forms small clusters beneath docked secretory granules, which are 50–100 nm in diameter and contain ~70 syntaxin molecules [47,53,59,60]. We confirmed that syntaxin clusters were readily observed and co-localized with docked granules (Fig 6A). During exocytosis of single granules, the associated syntaxin clusters rapidly dissolved (Fig 6A and 6B), as observed earlier [47,53]. To test whether syntaxin loss conincides with pore opening or content release, we used again non-linear fitting. The delay between content release and onset of syntaxin loss was -0.76±0.13 s, compared with only 0.11±0.16 s between pore opening and syntaxin loss (Fig 6D and 6E). Finally, all traces were temporally aligned to either the falling phase of NPY-td-mOrange2 (release) and averaged (Fig 6F). This analysis again shows that the syntaxin signal begins to decay before content release from the granule, while NPY-mTurquoise2 remains trapped until NPY-td-mOrange2 is released. Thus, statistical analysis (Fig 6D) and alignment of the traces (Fig 6B and 6E) indicate that loss of syntaxin coincides with the rising phase of td-mOrange2, suggesting that syntaxin dispersal is initiated already during pore formation. Experiments like this illustrate the utility pH-sensitive red FP’s for imaging the behavior of the exocytosis machinery.

## Discussion

In our survey of red FP’s utility to tag secretory granule cargo, we identified td-mOrange2 [41] as bright, photostable label that is efficiently targeted as fusion protein with NPY (see Table 1). As marker for secretory granule exocytosis, NPY-td-mOrange2 is comparable to the widely used NPY-mEGFP and other EGFP based fusion proteins and reports both fusion pore opening and content release due to its pH sensitivity. td-mOrange2 facilitates dual labeling experiments, in which exocytosis is studied together with other labeled proteins [30,32,33,34,41,53,61], sensors such as Ca^2+^-sensitive dyes (e.g. fluo-4), or knockdown approaches using fluorescently labeled siRNA, where it is often desirable or more convenient to use the red channel for a well-characterized organelle marker, and the green channel for other purposes. However, as suggested by its pKa, mOrange2 is much less sensitive to physiological pH changes than pHluorin.

**Table 1:** Features of NPY-FPs.

Construct	pKa	Brightness (relative to EGFP)	Fluorescence increase with NH_4_^+^	Bleaching rate (s^-1^)	Bleaching in presence of NH_4_^+^ (s^-1^)	Granule density (μm^-2^)	Non-granular structure density (μm^-2^)
NPY-mEGFP	6^[31]^	1.0±0.1	2.1±0.2	15.0±0.1	14.1±0.5	0.57±0.1	n.d.
NPY-pHluorin	7.6^[8]^	0.9±0.1	8.8±0.1	n.d.	14.7±0.4	n.d.	n.d.
NPY-mCherry	4.5^[38]^	0.8±0.1	1.0±0.1	6.1±0.3	n.d.	0.57±0.1	0.020±0.002
NPY-mOrange2	6.5^[5]^	1.2±0.1	1.3±0.1	5.8±0.3	6.1±0.8	0.59±0.1	0.020±0.004
NPY-td-mOrange2	6.5^[41]^	1.7±0.1	1.6±0.1	7.4±0.3	8.0±0.4	0.55±0.1	0.010±0.002
NPY-mApple	6.5^[5]^	1.1±0.1	1.3±0.1	3.9±0.2	4.1±0.2	0.55±0.1	0.010±0.002
NPY-mNectarine	6.9^[39]^	0.6±0.1	1.7±0.1	3.2±0.2	3.0±0.1	0.46±0.1	n.d.
NPY-mRuby2	5.3^[40]^	0.4±0.1	1.0±0.1	2.6±0.2	n.d.	0.53±0.1	n.d.
NPY-tdtomato	4.7^[38]^	1.7±0.1	1.1±0.1	8.0±0.3	n.d.	0.64±0.1	n.d.
NPY-pHtomato	7.8^[43]^	1.4±0.1	1.4±0.1	4.8±0.2	4.7±0.1	0.56±0.1	n.d.
NPY-pHuji	7.7^[28]^	0.7±0.1	1.5±0.1	6.7±0.3	6.9±0.1	0.42±0.1	0.06±0.01
NPY-TYG-Cherry	7.8^[28]^	0.6±0.1	1.9±0.1	9.7±0.2	13.4±0.4	0.36±0.1	0.10±0.01

In vitro, mOrange2 and tdTomato are among the brightest and slowest bleaching red FP’s [5]. Since this may not reflect the behavior in live cells, we compared brightness and photostability for red FP’s that were targeted to secretory granules. In intact granules, the td-tomato fusion was brightest, followed by td-mOrange2. However, given the 1.7-fold fluorescence increase upon neutralization suggests that NPY-td-mOrange2 is the brightest available red granule marker. Td-tomato and td-mOrange2 were somewhat more photostable than other tested red FP’s, which together with their improved brightness should enable long term imaging of vesicle behavior. Td-mOrange2 was originally created by duplexing mOrange2 [5] to facilitate detection of synaptic exo-and endocytosis [41]. For labeling small synaptic vesicles, the main advantage of the tandem dimer over monomeric mOrange2 is increased brightness, presumably due higher fluorophore density on the vesicles. We confirm here that granules labeled with td-mOrange2 are brighter than those labeled with the mOrange2 monomer, without changes in the release kinetics during exocytosis.

Most FPs tested here labeled at least 85% of the granuphilin positive granules, with the notable exceptions of pHuji and TYG-Cherry. Since granuphilin is soluble in the cytosol and bound to granules by weak interactions [62,63,64,65], it presumably labels all granules regardless of their history. NPY fusion constructs therefore lead to rapid, near quantitative labeling, which is important for many applications where knowledge about the absolute number of granules is required. However, mistargeting differed between the FP’s and the tandem dimer RFP fusions were generally better in this regard than the monomeric fusions, as judged by the lower density of large, rab7 labeled structures. Targeting accuracy of the tandem dimer to granules was comparable to that of EGFP, as was the fraction and density of labeled granules. We speculate that mistargeting results from protein aggregation at high luminal concentration, which is more prominent for the monomeric FPs than tandem dimers. Alternatively, lysosomal degradation of the fusion proteins might be more efficient for tandem dimers than monomeric versions, which leads to less accumulation within such structures. Steric effects as previously observed with dimers [5] do not appear to be a problem within secretory granules.

Green/Yellow FP’s with pKa>6 such as EGFP (pKa = 6) and pHluorin (pKa = 7.6) detect the luminal pH change during exocytosis and are therefore powerful tools to study membrane fusion and fission [9,10,24,30,34,66]. In (neuro)endocrine cells, dense core granules are large and sparse enough to be imaged individually and allow pH-sensitive FP’s to be used to detect the opening of the exocytotic fusion pore and to measure its life time [9,19,35,61]. Since EGFP retains some of its fluorescence in intact granules, it is a preferred granule marker as it allows observation and tracking of the intact organelles before exocytosis. In neurons, where synaptic vesicles are usually too small and too densely packed for individual observation, average signals from entire synapses are recorded. For this purpose, background suppression is important to increase the signal-to-noise ratio. PHluorin, which is non-fluorescent at the pH of intact synaptic vesicles, is therefore the protein of choice to detect neuronal exocytosis. However, red FP’s were originally developed to lack the often undesirable pH dependence [5,38,67], although some pH dependent variants have appeared (mOrange, mApple) [5]. Only recently, several novel pH-sensitive red FP’s have been specifically developed for imaging of endo- and exocytosis, including pHred [42], pHtomato [43] and pHuji [28]. Td-mOrange2 retains some of its fluorescence in intact granules. In contrast, pHuji is nearly non-fluorescent while in the lumen of some acidic vesicles [28], and is therefore expected to be an important complement to td-mOrange2. It is not clear why a significant fraction of the pHuji-labeled granules were bright before exocytosis (Fig 5B), while this was not observed with pHluorin. It is possible that these granules failed to acidify. Of the other pH dependent RFPs, mApple and mNectarine (reported pKa = 6.5 and 6.9, respectively; [5]) responded as expected to neutralizing luminal pH with NH_4_^+^, but were both dim and bleached rapidly. For reasons not understood, mApple did not detect the opening of the fusion pore during exocytosis.

In summary, we identify td-mOrange2 [41] as useful label for dense core granules; it is bright, well-targeted and spectrally separated from many blue and green FPs. It is likely suitable also for the study of many other membrane fusion or fission reactions, such as endocytosis and constitutive exocytosis.

## References

[1] Chalfie M, Tu Y, Euskirchen G, Ward WW, Prasher DC (1994) Green fluorescent protein as a marker for gene expression. Science 263: 802–805. 8303295 10.1126/science.8303295

[2] Prasher DC, Eckenrode VK, Ward WW, Prendergast FG, Cormier MJ (1992) Primary structure of the Aequorea victoria green-fluorescent protein. Gene 111: 229–233. 1347277 10.1016/0378-1119(92)90691-h

[3] Davidson MW, Campbell RE (2009) Engineered fluorescent proteins: innovations and applications. Nat Methods 6: 713–717. 19953681 10.1038/nmeth1009-713

[4] Kremers GJ, Gilbert SG, Cranfill PJ, Davidson MW, Piston DW (2011) Fluorescent proteins at a glance. J Cell Sci 124: 157–160. 10.1242/jcs.072744 21187342 PMC3037093

[5] Shaner NC, Lin MZ, McKeown MR, Steinbach PA, Hazelwood KL, et al (2008) Improving the photostability of bright monomeric orange and red fluorescent proteins. Nat Methods 5: 545–551. 10.1038/nmeth.1209 18454154 PMC2853173

[6] Schweizer FE, Betz H, Augustine GJ (1995) From vesicle docking to endocytosis: intermediate reactions of exocytosis. Neuron 14: 689–696. 7718232 10.1016/0896-6273(95)90213-9

[7] Steyer JA, Horstmann H, Almers W (1997) Transport, docking and exocytosis of single secretory granules in live chromaffin cells. Nature 388: 474–478. 9242406 10.1038/41329

[8] Sankaranarayanan S, Ryan TA (2000) Real-time measurements of vesicle-SNARE recycling in synapses of the central nervous system. Nat Cell Biol 2: 197–204. 10783237 10.1038/35008615

[9] Barg S, Olofsson CS, Schriever-Abeln J, Wendt A, Gebre-Medhin S, et al (2002) Delay between fusion pore opening and peptide release from large dense-core vesicles in neuroendocrine cells. Neuron 33: 287–299. 11804575 10.1016/s0896-6273(02)00563-9

[10] Miesenbock G, De Angelis DA, Rothman JE (1998) Visualizing secretion and synaptic transmission with pH-sensitive green fluorescent proteins. Nature 394: 192–195. 9671304 10.1038/28190

[11] Sankaranarayanan S, Ryan TA (2001) Calcium accelerates endocytosis of vSNAREs at hippocampal synapses. Nat Neurosci 4: 129–136. 11175872 10.1038/83949

[12] Gordon SL, Leube RE, Cousin MA (2011) Synaptophysin is required for synaptobrevin retrieval during synaptic vesicle endocytosis. J Neurosci 31: 14032–14036. 10.1523/JNEUROSCI.3162-11.2011 21957264 PMC3188371

[13] Hoppa MB, Lana B, Margas W, Dolphin AC, Ryan TA (2012) alpha2delta expression sets presynaptic calcium channel abundance and release probability. Nature 486: 122–125. 10.1038/nature11033 22678293 PMC3376018

[14] Wilhelm BG, Groemer TW, Rizzoli SO (2010) The same synaptic vesicles drive active and spontaneous release. Nat Neurosci 13: 1454–1456. 10.1038/nn.2690 21102450

[15] Hartman KN, Pal SK, Burrone J, Murthy VN (2006) Activity-dependent regulation of inhibitory synaptic transmission in hippocampal neurons. Nat Neurosci 9: 642–649. 16582905 10.1038/nn1677

[16] Hua Y, Sinha R, Thiel CS, Schmidt R, Huve J, et al (2011) A readily retrievable pool of synaptic vesicles. Nat Neurosci 14: 833–839. 10.1038/nn.2838 21666673

[17] Gandhi SP, Stevens CF (2003) Three modes of synaptic vesicular recycling revealed by single-vesicle imaging. Nature 423: 607–613. 12789331 10.1038/nature01677

[18] Ohara-Imaizumi M, Nakamichi Y, Tanaka T, Katsuta H, Ishida H, et al (2002) Monitoring of exocytosis and endocytosis of insulin secretory granules in the pancreatic beta-cell line MIN6 using pH-sensitive green fluorescent protein (pHluorin) and confocal laser microscopy. Biochem J 363: 73–80. 11903049 10.1042/0264-6021:3630073 PMC1222453

[19] Tsuboi T, Rutter GA (2003) Multiple forms of "kiss-and-run" exocytosis revealed by evanescent wave microscopy. Curr Biol 13: 563–567. 12676086 10.1016/s0960-9822(03)00176-3

[20] Xu Y, Rubin BR, Orme CM, Karpikov A, Yu C, et al (2011) Dual-mode of insulin action controls GLUT4 vesicle exocytosis. J Cell Biol 193: 643–653. 10.1083/jcb.201008135 21555461 PMC3166865

[21] Bai L, Zhu D, Zhou K, Zhou W, Li D, et al (2006) Differential properties of GTP- and Ca(2+)-stimulated exocytosis from large dense core vesicles. Traffic 7: 416–428. 16536740 10.1111/j.1600-0854.2006.00394.x

[22] Anantharam A, Onoa B, Edwards RH, Holz RW, Axelrod D (2010) Localized topological changes of the plasma membrane upon exocytosis visualized by polarized TIRFM. J Cell Biol 188: 415–428. 10.1083/jcb.200908010 20142424 PMC2819686

[23] Granseth B, Odermatt B, Royle SJ, Lagnado L (2006) Clathrin-mediated endocytosis is the dominant mechanism of vesicle retrieval at hippocampal synapses. Neuron 51: 773–786. 16982422 10.1016/j.neuron.2006.08.029

[24] Merrifield CJ, Perrais D, Zenisek D (2005) Coupling between clathrin-coated-pit invagination, cortactin recruitment, and membrane scission observed in live cells. Cell 121: 593–606. 15907472 10.1016/j.cell.2005.03.015

[25] Santos MS, Park CK, Foss SM, Li H, Voglmaier SM (2013) Sorting of the vesicular GABA transporter to functional vesicle pools by an atypical dileucine-like motif. J Neurosci 33: 10634–10646. 10.1523/JNEUROSCI.0329-13.2013 23804087 PMC3693053

[26] Xu J, Chai H, Ehinger K, Egan TM, Srinivasan R, et al (2014) Imaging P2X4 receptor subcellular distribution, trafficking, and regulation using P2X4-pHluorin. J Gen Physiol 144: 81–104. 10.1085/jgp.201411169 24935743 PMC4076521

[27] Jullie D, Choquet D, Perrais D (2014) Recycling endosomes undergo rapid closure of a fusion pore on exocytosis in neuronal dendrites. J Neurosci 34: 11106–11118. 10.1523/JNEUROSCI.0799-14.2014 25122907 PMC6705249

[28] Shen Y, Rosendale M, Campbell RE, Perrais D (2014) pHuji, a pH-sensitive red fluorescent protein for imaging of exo- and endocytosis. J Cell Biol 207: 419–432. 10.1083/jcb.201404107 25385186 PMC4226733

[29] Nagai T, Ibata K, Park ES, Kubota M, Mikoshiba K, et al (2002) A variant of yellow fluorescent protein with fast and efficient maturation for cell-biological applications. Nat Biotechnol 20: 87–90. 11753368 10.1038/nbt0102-87

[30] Taraska JW, Perrais D, Ohara-Imaizumi M, Nagamatsu S, Almers W (2003) Secretory granules are recaptured largely intact after stimulated exocytosis in cultured endocrine cells. Proc Natl Acad Sci U S A 100: 2070–2075. 12538853 10.1073/pnas.0337526100 PMC149960

[31] Yang TT, Cheng L, Kain SR (1996) Optimized codon usage and chromophore mutations provide enhanced sensitivity with the green fluorescent protein. Nucleic Acids Res 24: 4592–4593. 8948654 10.1093/nar/24.22.4592 PMC146266

[32] Felmy F (2009) Actin and dynamin recruitment and the lack thereof at exo- and endocytotic sites in PC12 cells. Pflugers Arch 458: 403–417. 10.1007/s00424-008-0623-1 19066940

[33] Perrais D, Kleppe IC, Taraska JW, Almers W (2004) Recapture after exocytosis causes differential retention of protein in granules of bovine chromaffin cells. J Physiol 560: 413–428. 15297569 10.1113/jphysiol.2004.064410 PMC1665250

[34] Tsuboi T, Zhao C, Terakawa S, Rutter GA (2000) Simultaneous evanescent wave imaging of insulin vesicle membrane and cargo during a single exocytotic event. Curr Biol 10: 1307–1310. 11069115 10.1016/s0960-9822(00)00756-9

[35] Michael DJ, Geng X, Cawley NX, Loh YP, Rhodes CJ, et al (2004) Fluorescent cargo proteins in pancreatic beta-cells: design determines secretion kinetics at exocytosis. Biophys J 87: L03–05. 15516519 10.1529/biophysj.104.052175 PMC1304941

[36] Sochacki KA, Larson BT, Sengupta DC, Daniels MP, Shtengel G, et al (2012) Imaging the post-fusion release and capture of a vesicle membrane protein. Nat Commun 3: 1154 10.1038/ncomms2158 23093191 PMC3521636

[37] Taylor MJ, Perrais D, Merrifield CJ (2011) A high precision survey of the molecular dynamics of mammalian clathrin-mediated endocytosis. PLoS Biol 9: e1000604 10.1371/journal.pbio.1000604 21445324 PMC3062526

[38] Shaner NC, Campbell RE, Steinbach PA, Giepmans BN, Palmer AE, et al (2004) Improved monomeric red, orange and yellow fluorescent proteins derived from Discosoma sp. red fluorescent protein. Nat Biotechnol 22: 1567–1572. 15558047 10.1038/nbt1037

[39] Johnson DE, Ai HW, Wong P, Young JD, Campbell RE, et al (2009) Red fluorescent protein pH biosensor to detect concentrative nucleoside transport. J Biol Chem 284: 20499–20511. 10.1074/jbc.M109.019042 19494110 PMC2742814

[40] Lam AJ, St-Pierre F, Gong Y, Marshall JD, Cranfill PJ, et al (2012) Improving FRET dynamic range with bright green and red fluorescent proteins. Nat Methods 9: 1005–1012. 10.1038/nmeth.2171 22961245 PMC3461113

[41] Li H, Foss SM, Dobryy YL, Park CK, Hires SA, et al (2011) Concurrent imaging of synaptic vesicle recycling and calcium dynamics. Front Mol Neurosci 4: 34 10.3389/fnmol.2011.00034 22065946 PMC3206542

[42] Tantama M, Hung YP, Yellen G (2011) Imaging intracellular pH in live cells with a genetically encoded red fluorescent protein sensor. J Am Chem Soc 133: 10034–10037. 10.1021/ja202902d 21631110 PMC3126897

[43] Li Y, Tsien RW (2012) pHTomato, a red, genetically encoded indicator that enables multiplex interrogation of synaptic activity. Nat Neurosci 15: 1047–1053. 10.1038/nn.3126 22634730 PMC3959862

[44] Lampe M, Pierre F, Al-Sabah S, Krasel C, Merrifield CJ (2014) Dual single-scission event analysis of constitutive transferrin receptor (TfR) endocytosis and ligand-triggered beta2-adrenergic receptor (beta2AR) or Mu-opioid receptor (MOR) endocytosis. Mol Biol Cell 25: 3070–3080. 10.1091/mbc.E14-06-1112 25079691 PMC4230595

[45] Hohmeier HE, Mulder H, Chen G, Henkel-Rieger R, Prentki M, et al (2000) Isolation of INS-1-derived cell lines with robust ATP-sensitive K+ channel-dependent and-independent glucose-stimulated insulin secretion. Diabetes 49: 424–430. 10868964 10.2337/diabetes.49.3.424

[46] Lang T, Wacker I, Steyer J, Kaether C, Wunderlich I, et al (1997) Ca2+-triggered peptide secretion in single cells imaged with green fluorescent protein and evanescent-wave microscopy. Neuron 18: 857–863. 9208853 10.1016/s0896-6273(00)80325-6

[47] Barg S, Knowles MK, Chen X, Midorikawa M, Almers W (2010) Syntaxin clusters assemble reversibly at sites of secretory granules in live cells. Proc Natl Acad Sci U S A 107: 20804–20809. 10.1073/pnas.1014823107 21076041 PMC2996446

[48] Galvez-Santisteban M, Rodriguez-Fraticelli AE, Bryant DM, Vergarajauregui S, Yasuda T, et al (2012) Synaptotagmin-like proteins control the formation of a single apical membrane domain in epithelial cells. Nat Cell Biol 14: 838–849. 10.1038/ncb2541 22820376 PMC3433678

[49] Jordens I, Westbroek W, Marsman M, Rocha N, Mommaas M, et al (2006) Rab7 and Rab27a control two motor protein activities involved in melanosomal transport. Pigment Cell Res 19: 412–423. 16965270 10.1111/j.1600-0749.2006.00329.x

[50] Goedhart J, von Stetten D, Noirclerc-Savoye M, Lelimousin M, Joosen L, et al (2012) Structure-guided evolution of cyan fluorescent proteins towards a quantum yield of 93%. Nat Commun 3: 751 10.1038/ncomms1738 22434194 PMC3316892

[51] Spiliotis M (2012) Inverse fusion PCR cloning. PLoS One 7: e35407 10.1371/journal.pone.0035407 22530019 PMC3328455

[52] Torii S, Takeuchi T, Nagamatsu S, Izumi T (2004) Rab27 effector granuphilin promotes the plasma membrane targeting of insulin granules via interaction with syntaxin 1a. J Biol Chem 279: 22532–22538. 15028737 10.1074/jbc.M400600200

[53] Gandasi NR, Barg S (2014) Contact-induced clustering of syntaxin and munc18 docks secretory granules at the exocytosis site. Nat Commun 5: 3914 10.1038/ncomms4914 24835618

[54] Snaith HA, Anders A, Samejima I, Sawin KE (2010) New and old reagents for fluorescent protein tagging of microtubules in fission yeast; experimental and critical evaluation. Methods Cell Biol 97: 147–172. 10.1016/S0091-679X(10)97009-X 20719270

[55] Vanlandingham PA, Ceresa BP (2009) Rab7 regulates late endocytic trafficking downstream of multivesicular body biogenesis and cargo sequestration. J Biol Chem 284: 12110–12124. 10.1074/jbc.M809277200 19265192 PMC2673280

[56] Vonderheit A, Helenius A (2005) Rab7 associates with early endosomes to mediate sorting and transport of Semliki forest virus to late endosomes. PLoS Biol 3: e233 15954801 10.1371/journal.pbio.0030233 PMC1151600

[57] Jahn R, Lang T, Sudhof TC (2003) Membrane fusion. Cell 112: 519–533. 12600315 10.1016/s0092-8674(03)00112-0

[58] Ohara-Imaizumi M, Fujiwara T, Nakamichi Y, Okamura T, Akimoto Y, et al (2007) Imaging analysis reveals mechanistic differences between first- and second-phase insulin exocytosis. J Cell Biol 177: 695–705. 17502420 10.1083/jcb.200608132 PMC2064214

[59] Sieber JJ, Willig KI, Kutzner C, Gerding-Reimers C, Harke B, et al (2007) Anatomy and dynamics of a supramolecular membrane protein cluster. Science 317: 1072–1076. 17717182 10.1126/science.1141727

[60] Lang T (2007) SNARE proteins and 'membrane rafts'. J Physiol 585: 693–698. 17478530 10.1113/jphysiol.2007.134346 PMC2375502

[61] Obermuller S, Lindqvist A, Karanauskaite J, Galvanovskis J, Rorsman P, et al (2005) Selective nucleotide-release from dense-core granules in insulin-secreting cells. Journal of Cell Science 118: 4271–4282. 16141231 10.1242/jcs.02549

[62] Coppola T, Frantz C, Perret-Menoud V, Gattesco S, Hirling H, et al (2002) Pancreatic beta-cell protein granuphilin binds Rab3 and Munc-18 and controls exocytosis. Mol Biol Cell 13: 1906–1915. 12058058 10.1091/mbc.02-02-0025 PMC117613

[63] Gomi H, Mizutani S, Kasai K, Itohara S, Izumi T (2005) Granuphilin molecularly docks insulin granules to the fusion machinery. J Cell Biol 171: 99–109. 16216924 10.1083/jcb.200505179 PMC2171228

[64] Handley MT, Burgoyne RD (2008) The Rab27 effector Rabphilin, unlike Granuphilin and Noc2, rapidly exchanges between secretory granules and cytosol in PC12 cells. Biochem Biophys Res Commun 373: 275–281. 10.1016/j.bbrc.2008.06.043 18573236

[65] Torii S, Zhao S, Yi Z, Takeuchi T, Izumi T (2002) Granuphilin modulates the exocytosis of secretory granules through interaction with syntaxin 1a. Mol Cell Biol 22: 5518–5526. 12101244 10.1128/MCB.22.15.5518-5526.2002 PMC133943

[66] Perrais D, Merrifield CJ (2005) Dynamics of endocytic vesicle creation. Dev Cell 9: 581–592. 16256734 10.1016/j.devcel.2005.10.002

[67] Subach FV, Patterson GH, Renz M, Lippincott-Schwartz J, Verkhusha VV (2010) Bright monomeric photoactivatable red fluorescent protein for two-color super-resolution sptPALM of live cells. J Am Chem Soc 132: 6481–6491. 10.1021/ja100906g 20394363 PMC2866019

